# Diagnosing pancreatic neuroendocrine tumors in patients with multiple endocrine neoplasia type 1 in daily practice

**DOI:** 10.3389/fendo.2022.926491

**Published:** 2022-10-07

**Authors:** Dirk-Jan van Beek, Carolina R. C. Pieterman, Frank J. Wessels, Annenienke C. van de Ven, Wouter W. de Herder, Olaf M. Dekkers, Wouter T. Zandee, Madeleine L. Drent, Peter H. Bisschop, Bas Havekes, Inne H. M. Borel Rinkes, Menno R. Vriens, Gerlof D. Valk

**Affiliations:** ^1^ Department of Endocrine Surgical Oncology, University Medical Center Utrecht, Utrecht, Netherlands; ^2^ Department of Endocrine Oncology, University Medical Center Utrecht, Utrecht, Netherlands; ^3^ Department of Radiology, University Medical Center Utrecht, Utrecht, Netherlands; ^4^ Department of Endocrinology, Radboud University Medical Center, Nijmegen, Netherlands; ^5^ Department of Internal Medicine, Erasmus Medical Center, Rotterdam, Netherlands; ^6^ Departments of Endocrinology and Metabolism and Clinical Epidemiology, Leiden University Medical Center, Leiden, Netherlands; ^7^ Department of Endocrinology, University of Groningen, University Medical Center Groningen, Groningen, Netherlands; ^8^ Department of Internal Medicine, Section of Endocrinology, Amsterdam University Medical Center (UMC) Location Vrije Universiteit (VU) University Medical Center, Amsterdam, Netherlands; ^9^ Department of Endocrinology and Metabolism, Amsterdam University Medical Center (UMC) Location Academic Medical Center, Amsterdam, Netherlands; ^10^ Department of Internal Medicine, Division of Endocrinology, Maastricht University Medical Center, Maastricht, Netherlands

**Keywords:** multiple endocrine neoplasia type 1, pancreatic neuroendocrine tumor, imaging, MRI, CT, EUS, FNA, diagnosis

## Abstract

**Background:**

In multiple endocrine neoplasia type 1 (MEN1), pancreatic neuroendocrine tumors (PanNETs) have a high prevalence and represent the main cause of death. This study aimed to assess the diagnostic accuracy of the currently used conventional pancreatic imaging techniques and the added value of fine needle aspirations (FNAs).

**Methods:**

Patients who had at least one imaging study were included from the population-based MEN1 database of the DutchMEN Study Group from 1990 to 2017. Magnetic resonance imaging (MRI), computed tomography (CT), endoscopic ultrasonography (EUS), FNA, and surgical resection specimens were obtained. The first MRI, CT, or EUS was considered as the index test. For a comparison of the diagnostic accuracy of MRI versus CT, patients with their index test taken between 2010 and 2017 were included. The reference standard consisted of surgical histopathology or radiological follow-up.

**Results:**

A total of 413 patients (92.8% of the database) underwent 3,477 imaging studies. The number of imaging studies per patient increased, and a preference for MRI was observed in the last decade. Overall diagnostic accuracy was good with a positive (PPV) and negative predictive value (NPV) of 88.9% (95% confidence interval, 76.0–95.6) and 92.8% (89.4–95.1), respectively, for PanNET in the pancreatic head and 92.0% (85.3–96.0) and 85.3% (80.5–89.1), respectively, in the body/tail. For MRI, PPV and NPV for pancreatic head tumors were 100% (76.1–100) and 87.1% (76.3–93.6) and for CT, 60.0% (22.9–88.4) and 70.4% (51.3–84.3), respectively. For body/tail tumors, PPV and NPV were 91.3% (72.0–98.8) and 87.0% (75.3–93.9), respectively, for MRI and 100% (74.9–100) and 77.8% (54.3–91.5), respectively, for CT. Pathology confirmed a PanNET in 106 out of 110 (96.4%) resection specimens. FNA was performed on 34 lesions in 33 patients and was considered PanNET in 24 [all confirmed PanNET by histology (10) or follow-up (14)], normal/cyst/unrepresentative in 6 (all confirmed PanNET by follow-up), and adenocarcinoma in 4 (2 confirmed and 2 PanNET). Three patients, all older than 60 years, had a final diagnosis of pancreatic adenocarcinoma.

**Conclusion:**

As the accuracy for diagnosing MEN1-related PanNET of MRI was higher than that of CT, MRI should be the preferred (non-invasive) imaging modality for PanNET screening/surveillance. The high diagnostic accuracy of pancreatic imaging and the sporadic occurrence of pancreatic adenocarcinoma question the need for routine (EUS-guided) FNA.

## Introduction

Multiple endocrine neoplasia type 1 (MEN1) is an autosomal dominant tumor syndrome with an estimated prevalence of 1–10 patients per 100,000 people (1, 2). Primary hyperparathyroidism, duodenopancreatic neuroendocrine tumors (dpNETs), and pituitary adenomas are the clinical hallmarks of the syndrome (3). During their lifetime, more than 80% of patients are affected by dpNETs, and metastasized dpNETs represent the leading cause of death (4–6).

To enable timely diagnosis of pancreatic neuroendocrine tumors (PanNETs), MEN1 clinical practice guidelines suggest yearly conventional pancreatic imaging with computed tomography (CT), magnetic resonance imaging (MRI), or endoscopic ultrasonography (EUS) (7). In a previous systematic review, the diagnostic accuracy of pancreatic imaging in MEN1 was found to be relatively high (8). Consequently, a strategy combining MRI and EUS for the diagnosis of NF-PanNETs was advised, however, with remaining uncertainty about the optimal modality (8). Most included studies reported on EUS or CT only, were mainly single center, and were methodologically heterogeneous with varying risks of bias (8). More importantly, only one study compared MRI and CT; only eight patients had an MRI, and all underwent operative resection (9). As such, firm conclusions regarding a preferred non-invasive imaging modality could not be drawn (8). Diagnostic accuracy measures derived from cohorts including patients with sporadically occurring PanNET cannot be extrapolated to MEN1, since in sporadic PanNETs, imaging is used for case finding, whereas in MEN1, it is used for screening. Additionally, the higher prior probability of PanNETs due to the underlying germline mutation in patients with MEN1 should be taken into account.

Given the high prior probability for a PanNET in patients with MEN1, once a suspicious pancreatic lesion is identified, it is unclear if histopathological confirmation is necessary. For sporadic non-functioning PanNETs, the European Neuroendocrine Tumor Society Guidelines suggest EUS-guided biopsies, but no specific recommendations are proposed for patients with MEN1 (10). A recent consensus statement lacked a conclusion regarding if and when EUS-guided biopsies are to be performed for the diagnosis of MEN1-related PanNETs (11). However, when the diagnostic accuracy of pancreatic imaging is proven to be high, the added value of pancreatic biopsies for diagnostic purposes may be questioned.

Hence, the aims of the present study were to assess the diagnostic accuracy of conventional pancreatic imaging studies and to determine the added value of pancreatic fine needle aspiration (FNA) for the diagnosis of MEN1-related PanNETs.

## Methods

Reporting of the study was performed according to the STAndards for the Reporting of Diagnostic accuracy studies (STARD) checklist (12).

### Study design—DMSG database

Patients with at least one conventional pancreatic imaging were included in the present study from the DutchMEN Study Group (DMSG) database, which has been described previously (13). Briefly, patients with MEN1 aged 16 years and older and followed in one of the eight Dutch University Medical Centers (UMC) are included. Within each center, patients were identified by medical conditions and disease review of hospital databases. The MEN1 diagnosis was established according to the Clinical Practice Guidelines for MEN1 (7). Over 90% of the Dutch MEN1 population is included in the database. Clinical and demographic data were collected longitudinally every quarter from 1990 to 2014 by standardized medical record review, according to a predefined protocol. From 2014 onwards, data were captured prospectively. The protocol was approved by the Medical Ethics Committees of all UMCs.

### Imaging studies and pathology in the DMSG database

All pancreatic imaging studies (CT, MRI, or EUS) in the setting of screening and surveillance for MEN1-related manifestations from January 1 1990 until 31 December 2017 were captured in the database. Imaging studies were captured per quarter of each year; if multiple investigations of the same modality were performed within the same quarter, only the first examination was entered. Imaging studies with other aims, e.g., to detect postoperative complications, were disregarded. Data were collected from routine patient care. The original reports were used; no review of images was performed. Radiologists, gastroenterologists, and pathologists were not blinded to previous imaging or to clinical information. According to the guidelines, outcomes were discussed in multidisciplinary tumor boards within the individual centers; conclusions could subsequently be altered based on these discussions (7).

### Index tests

#### I. Diagnostic accuracy of conventional pancreatic imaging, irrespective type of imaging, in MEN1 patients under surveillance from 1990 to 2017

For each patient, the index test was defined as the first conventional screening/surveillance imaging that was performed on the pancreas. This could be either CT, MRI, or EUS. The pancreatic head and pancreatic body/tail were analyzed separately. One dominant (largest) tumor from the pancreatic head and one from the pancreatic body or tail were analyzed.

#### II. Diagnostic accuracy of CT versus MRI

For the specific comparison of the diagnostic accuracy between MRI and CT, data from patients whose first pancreatic screening/surveillance imaging was conducted between 2010 and 2017 and was either an MRI or CT were analyzed. The index test was the first pancreatic screening/surveillance by MRI or CT in this time period. Comparison groups were defined based on the imaging modality of the index tests (MRI or CT).

Patients with a PanNET diagnosis before 1990 were not considered for the imaging analyses.

### Reference standard

The reference test was histopathology (surgical resection) or radiological follow-up. Histological verification in all patients was not possible because not everyone underwent a resection. In the absence of histopathology, repetitive follow-up was used as reference test (14, 15). To determine the outcome of the reference test in those with only radiological follow-up, the follow-up conventional imaging within 3 years of the index test was used; this could be either CT/MRI or EUS independent of the modality of the original index text. If the first follow-up conventional imaging after the index test was positive but followed by two negative imaging studies within 3 years without operative resection, the reference test was negative (i.e., the patient was deemed not to have a PanNET) (16). The size of the PanNET on follow-up imaging was also taken into account; any follow-up scan within 3 years of the index test that showed a PanNET of 5 mm or larger was considered a positive reference test (conditional on not being followed by two negative tests as described above). Based on reported PanNET growth rates (0.1–1.32 mm/year) in this study population, it was hypothesized that a tumor of 5 mm or larger should have been visible on imaging studies within the prior 3 years (8, 16).

### Resection specimens and FNA

All resection specimens and EUS-guided pancreatic FNA after 1990 were retrieved from the database, regardless of a PanNET diagnosis before 1990. Resection specimens and FNAs were assessed for the presence of a PanNET, and possible other outcomes were captured. Negative or inconclusive biopsies were classified as such. In those with an FNA and subsequent resection specimen, tumor grade was classified according to the World Health Organization (WHO) 2017 classification: Grade 1 (G1), Ki67 labeling index (LI) <3 and mitosis <2 per 10 high power fields (HPF); G2, Ki67 LI of 3–20 and/or mitoses of 2–20/10 HPF; G3, Ki67 LI >20 and/or mitosis >20/10 HPF (17).

### Statistical analysis

Descriptive statistics were reported as mean ± standard deviation (SD) or median [interquartile range (IQR) or range] for continuous variables or as counts (percentages) for categorical variables. The average number of imaging studies per 100 patients per year was calculated.

Outcomes were diagnostic accuracy measures, sensitivity, specificity, positive predictive value (PPV), negative predictive value (NPV), positive likelihood ratio (LR), and negative LR. Binomial Agresti–Coull 95% confidence intervals (CIs) were calculated for sensitivity, specificity, PPV, and NPV (18). Likelihood ratio 95% CIs were defined based on the log method (19). Subgroup analyses according to the time period (1990–1999, 2000–2009, and 2010–2017) and the reference standard (histopathology *vs*. imaging follow-up) were performed.

Statistical analyses were performed by using SPSS version 25.0 (IBM Corp, Armonk, NY) and R version 3.5.1 (R Foundation for Statistical Computing, Vienna, Austria). Figures were constructed by using GraphPad Prism version 7.02 (GraphPad Software, Inc., San Diego, CA).

## Results

### DMSG database

A total of 445 patients were identified in the database, of whom 413 patients (92.8%) underwent at least one pancreatic imaging study. In total, 3,477 imaging studies were performed, of which 1,818 (52.3%) were MRI, 1,291 (37.1%) were CT, and 368 (10.6%) were EUS, respectively (**Supplementary Table S1**). In 1,845 (53.1%) studies, a PanNET was reported. Median radiological follow-up time from the first scan after 1990 was 8.4 years (IQR, 4.5–13.8). Twenty-nine patients (6.5%) were lost to follow-up. The mean age at the last date of follow-up was 50.6 years ( ± 16.6).

### Imaging over time

**Figure 1:**
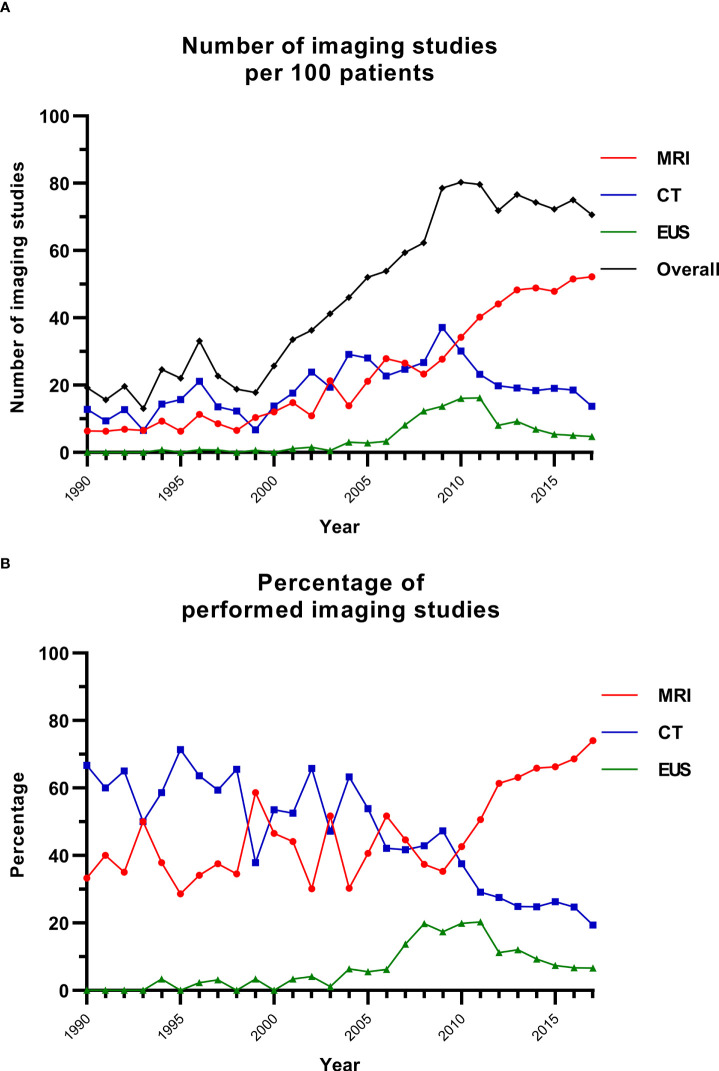
Imaging studies over time. **(A)** The number of imaging studies over time per 100 patients. **(B)** The percentage of imaging studies for each modality per year. CT, computed tomography; EUS, endoscopic ultrasonography; MRI, magnetic resonance imaging.

Since 2009, between 70 and 80 imaging studies were conducted per 100 patients in the database annually (**Figure 1**). The contribution of MRI increased from 38.4% of all performed imaging in 1990–1999 (range per year, 28.6%–58.6%) to 61.4% in 2010–2017 (range per year, 42.6%–74.0%) (**Figure 1**; **Supplementary Table S1**). The relative use of CT decreased from 60.0% to 26.9% and that of EUS increased from 1.6% to 11.8%.

The percentage of positive index scans was 39.4% in 1990–1999, 30.1% in 2000–2009, and 37.0% in 2010–2017. Of the patients with MRI as index test, 33.7% had a positive index MRI in 2010–2017, 19.8% in 2000–2009, and 38.7% in 1990–1999 (**Supplementary Table S1**). For CT, these percentages were 40.0%, 37.0%, and 44.4%, respectively. When stratifying by age (10-year strata), the increase in the percentage of positive studies increased with age at the index scan (**Supplementary Table S2**).

### Diagnostic accuracy of all conventional imaging

Of the 413 patients with imaging studies, 17 patients (4.1%) had a PanNET diagnosis before 1990, and 19 patients (4.6%) had only one imaging study without resection. Subsequently, 377 patients were available for analysis; their mean age at the index study was 38.6 ( ± 15.8) years. A total of 131 patients (34.7%) had a positive index test, of whom 26 had both a PanNET of the pancreatic head and body/tail. Forty-five index studies documented a PanNET of the pancreatic head, of which 40 (88.9%) were considered as PanNET by the reference standard, and 112 index studies reported a PanNET of the body/tail, of which 103 (92.0%) were considered as PanNET by the reference standard. MRI and CT were the index study in 192 (50.9%) and 184 (48.8%) patients, respectively, whereas only one patient had EUS as index test.

Contingency tables are presented in **Table 1**, and diagnostic accuracy is reported in **Table 2**. The diagnostic accuracy of all conventional imaging, irrespective of the type of imaging, for tumors in the pancreatic head was sensitivity of 62.5% (50.2–73.4), specificity of 98.4% (96.2–99.4), PPV of 88.9% (76.0–95.6), and NPV of 92.8% (89.4–95.1), respectively. For pancreatic body/tail tumors, sensitivity was 72.5% (64.6–79.2), specificity was 96.2% (92.8–98.1), PPV was 92.0% (85.3–96.0), and NPV was 85.3% (80.5–89.1), respectively.

**Table 1:** Contingency tables of all imaging studies 1990–2017.

Pancreatic head				Pancreatic body/tail			
	Reference standard				Reference standard		
	**PanNET (no, %)**	**No PanNET (no, %)**			**PanNET (no, %)**	**No PanNET (no, %)**	
**All imaging (n = 377)**			Total	**All imaging (n = 377)**			Total
Index PanNET	40 (62.5)	5 (1.6)	45 (11.9)	Index PanNET	103 (72.5)	9 (3.8)	112 (29.7)
Index no PanNET	24 (37.5)	308 (98.4)	332 (88.1)	Index no PanNET	39 (27.5)	226 (96.2)	265 (70.3)
Total	64 (17.0)	313 (83.0)	377 (100)	Total	142 (37.7)	235 (62.3)	377 (100)
**2010–2017 (n = 109)**			Total	**2010–2017 (n = 109)**			Total
Index PanNET	18 (52.9)	2 (2.7)	20 (18.3)	Index PanNET	35 (76.1)	2 (3.2)	37 (33.9)
Index no PanNET	16 (47.1)	73 (97.3)	89 (81.7)	Index no PanNET	11 (23.9)	61 (96.8)	72 (66.1)
Total	34 (31.2)	75 (68.8)	109 (100)	Total	46 (42.2)	63 (57.8)	109 (100)
**2000–2009 (n = 199)**			Total	**2000–2009 (n = 199)**			Total
Index PanNET	17 (70.8)	1 (0.6)	18 (9.0)	Index PanNET	51 (71.8)	1 (0.8)	52 (26.1)
Index no PanNET	7 (29.2)	174 (99.4)	181 (91.0)	Index no PanNET	20 (28.6)	127 (99.2)	147 (73.9)
Total	24 (12.1)	175 (87.9)	199 (100)	Total	71 (35.7)	128 (64.3)	199 (100)
**1990–1999 (n = 69)**			Total	**1990–1999 (n = 69)**			Total
Index PanNET	5 (83.3)	2 (3.2)	7 (10.1)	Index PanNET	17 (68.0)	6 (13.6)	23 (33.3)
Index no PanNET	1 (16.7)	61 (96.8)	62 (89.9)	Index no PanNET	8 (32.0)	38 (86.4)	46 (66.7)
Total	6 (8.7)	63 (91.3)	69 (100)	Total	25 (36.2)	44 (63.8)	69 (100)

In the 2×2 tables, percentages are column percentages. The row “total” has row percentages; the column “total” has column percentages.

PanNET, pancreatic neuroendocrine tumor.

95% CIs are given in parentheses.

LR, likelihood ratio; NPV, negative predictive value; PPV, positive predictive value.

**Table 2:** Diagnostic accuracy of conventional imaging (CT/MRI/EUS) 1990–2017.

	Sensitivity	Specificity	PPV	NPV	Positive LR	Negative LR
**Pancreatic head**						
**Conventional imaging 1990–2017**	62.5 (50.2–73.4)	98.4 (96.2–99.4)	88.9 (76.0–95.6)	92.8 (89.4–95.1)	39.1 (16.1–95.3)	0.38 (0.28–0.52)
**2010–2017**	52.9 (36.7–68.6)	97.3 (90.2–99.8)	90.0 (68.7–98.4)	82.0 (72.7–88.7)	19.9 (4.9–80.8)	0.48 (0.34–0.70)
**2000–2009**	70.8 (50.6–85.3)	99.4 (96.5–100)	94.4 (72.4–100)	96.1 (92.1–98.3)	124.0 (17.3–889.9)	0.29 (0.16–0.55)
**1990–1999**	83.3 (41.8–98.9)	96.8 (88.5–99.8)	71.4 (35.2–92.4)	98.4 (90.6–100)	26.3 (6.4–107.5)	0.17 (0.03–1.03)
**Pancreatic body/tail**						
**Conventional imaging 1990–2017**	72.5 (64.6–79.2)	96.2 (92.8–98.1)	92.0 (85.3–96.0)	85.3 (80.5–89.1)	18.9 (9.9–36.2)	0.29 (0.22–0.37)
**2010–2017**	76.1 (61.9–86.2)	96.8 (88.5–99.8)	94.6 (81.4–99.4)	84.7 (74.5–91.4)	24.0 (6.1–94.6)	0.25 (0.15–0.41)
**2000–2009**	71.8 (60.4–81.0)	99.2 (95.3–100)	98.1 (88.9–100)	86.4 (79.8–91.1)	91.9 (13.0–651.3)	0.28 (0.20–0.41)
**1990–1999**	68.0 (48.3–82.9)	86.4 (72.9–94.0)	73.9 (53.2–87.7)	82.6 (69.0–91.2)	5.0 (2.3–11.0)	0.37 (0.21–0.66)

95% CIs are given in parentheses.

LR, likelihood ratio; NPV, negative predictive value; PPV, positive predictive value.

In total, 90 potential PanNETs had histopathology as reference standard, of which 20 (22.2%) were not reported on the index test (**Supplementary Table S3**). Of the 15 patients who underwent operative resection within 2 years of the index test, the median histological tumor size was 12 mm (range, 6–29). Sensitivity analyses according to the applied reference standard are reported in **Supplementary Table S4**.

### Diagnostic accuracy of CT and MRI

A total of 109 patients (26.4%) had their first CT or MRI between 2010 and 2017, of whom 77 (70.6%) had MRI and 32 (29.4%) had CT as initial imaging study. The median age at the first imaging study was 39.0 years (IQR, 19.2–54.1). The index test was positive for a PanNET in 43 patients (39.4%), and 56 patients (51.4%) had a PanNET according to the reference standard (**Table 3**). Median radiological size for the pancreatic head and body/tail tumors were 9 mm (IQR, 7–11) and 12 mm (IQR, 9–17.5), respectively. Histopathology was part of the reference standard in 6 patients (5.5%) with a PanNET in the pancreatic head and 16 patients (14.7%) with a PanNET in body/tail.

**Table 3:** Contingency tables of MRI and CT as first imaging study in 2010–2017.

Pancreatic head				Pancreatic body/tail			
	Reference standard				Reference standard		
**PanNET (no, %)**	**No PanNET (no, %)**			**PanNET (no, %)**	**No PanNET (no, %)**	
**MRI (n = 77)**			Total	**MRI (n = 77)**			Total
Index positive	15 (65.2)	0 (0)	15 (19.5)	Index positive	21 (75.0)	2 (4.1)	23 (29.9)
Index negative	8 (34.8)	54 (100)	62 (80.5)	Index negative	7 (25.0)	47 (95.9)	54 (70.1)
Total	23 (29.9)	54 (70.1)	77 (100)	Total	28 (36.4)	49 (63.6)	77 (100)
**CT (n = 32)**			Total	**CT (n = 32)**			Total
Index positive	3 (27.3)	2 (9.5)	5 (15.6)	Index positive	14 (77.8)	0 (0)	14 (43.8)
Index negative	8 (72.7)	19 (90.5)	27 (84.4)	Index negative	4 (22.2)	14 (100)	18 (56.3)
Total	11 (34.4)	21 (65.6)	32 (100)	Total	18 (56.3)	14 (43.8)	32 (100)

In the 2×2 tables, percentages are column percentages. The row “total” has row percentages; the column “total” has column percentages.

CT, computed tomography; MRI, magnetic resonance imaging; PanNET, pancreatic neuroendocrine tumor.

Contingency tables (**Table 3**) show that MRI was true positive for 15 out of 15 pancreatic head tumors and 21 out of 23 pancreatic body/tail tumors, indicating a PPV of 100% (76.1–100) for tumors in the head and 91.3% (72.0–98.8) for tumors in the body/tail (**Table 4**). For CT, 3 of 5 were positive in the pancreatic head and 14 out of 14 in the pancreatic body/tail. The corresponding PPV for CT was 60% (22.9–88.4) and 100% (74.9–100), respectively. For the pancreatic head, 8 of 27 patients with a negative CT were considered to have a PanNET [NPV, 70.4% (51.3–84.3)], compared with 8 of 62 patients with a negative MRI [NPV, 87.1% (76.3–93.6)], respectively. For the pancreatic body/tail, 4 of 14 patients with a negative CT were deemed to have a PanNET [NPV, 77.8% (54.3–91.5)] versus 7 of 54 patients with a negative MRI [NPV, 87.0% (75.3–93.9)].

**Table 4:** Diagnostic accuracy measures of MRI and CT as first imaging study in 2010–2017.

	Sensitivity	Specificity	PPV	NPV	Positive LR	Negative LR
**Pancreatic head**						
**MRI**	65.2 (44.8–81.3)	100 (92.1–100)	100 (76.1–100)	87.1 (76.3–93.6)	Inf	0.35 (0.20–0.61)
**CT**	27.3 (9.2–57.1)	90.5 (69.9–98.6)	60.0 (22.9–88.4)	70.4 (51.3–84.3)	2.86 (0.56–14.7)	0.80 (0.55–1.18)
**Pancreatic body/tail**						
**MRI**	75.0 (56.4–87.6)	95.9 (85.5–99.6)	91.3 (72.0–98.8)	87.0 (75.3–93.9)	18.4 (4.7 – 72.6)	0.26 (0.14–0.50)
**CT**	77.8 (54.3–91.5)	100 (74.9–100)	100 (74.9–100)	77.8 (54.3–91.5)	Inf	0.22 (0.09–0.53)

95% CIs are given in parentheses.

CT, computed tomography; LR, likelihood ratio; MRI, magnetic resonance imaging; NPV, negative predictive value; PPV, positive predictive value.

For both modalities, diagnostic accuracy measures were generally similar for the pancreatic head and body/tail (**Table 4**). For CT, lower sensitivity and PPV for pancreatic head tumors, albeit with wide confidence intervals, were observed. For tumors in the pancreatic head, point estimates of sensitivity, PPV, and NPV were higher for MRI, but 95% CIs overlapped. For the pancreatic body/tail, diagnostic accuracy measures were similar between both modalities.

### Resection specimens

A total of 110 surgical resection specimens were available, of which 106 (96.4%) had a final diagnosis of PanNET (**Table 5**). Two patients were considered to have a pancreatic ductal adenocarcinoma (PDAC); one of those underwent operative resection of a collision tumor consisting of a PDAC and surrounding PanNETs. Two patients had normal pancreatic tissue after resection. The first patient underwent an enucleation of the pancreatic body showing normal pancreatic tissue; follow-up imaging studies showed a 14-mm tumor. The other patient underwent an unsuccessful distal pancreatectomy without PanNET followed by a successful resection of the tumor during follow-up.

**Table 5:** Histopathological outcomes of all specimens.

	All specimens	Resection specimen	FNA
PanNET	131 (89.1%)^*^	106 (96.4%)^*^	25 (67.6%)
Normal pancreas	6 (4.1%)	2 (1.8%)	4 (10.8%)
PDAC	7 (4.8%)^*|^^	2 (1.8%)^*^	5 (13.5%)^|^^
Other or not representative	3 (2.0%)	0 (0%)	3 (8.1%)

All tissues were collected indicating that patients could contribute multiple specimens; with regards to FNA, three patients had two biopsies of the same lesion. In one patient, the FNA was positive for a PanNET twice; in one, normal pancreatic tissue was followed by PanNET; and in the last patient, the biopsy was positive for PDAC twice. One patient had FNA of two lesions, so the 37 FNA results represent 34 lesions from 22 patients.

^*^In one patient, the tumor was a collision tumor consisting of a PDAC surrounded by multiple PanNETs.

^|^In one patient, a biopsy was initially positive for PDAC; revision of the specimen during follow-up changed the conclusion into a PanNET.

^^^In one patient with a PDAC-positive biopsy, the surgical resection specimen revealed a PanNET and no PDAC.

FNA, fine needle aspiration; PDAC, pancreatic ductal adenocarcinoma; PanNET, pancreatic neuroendocrine tumor.

### Pancreatic FNA

In total, FNA was performed on 34 pancreatic lesions in 33 patients; in three lesions, FNA was performed twice. This means that a total of 37 FNA results were available for 33 patients (**Table 5**). FNA was obtained once in 29 patients and twice in 4 patients. In the three patients with FNA of the same lesion more than once, the FNA was positive for PanNET twice in one patient, normal pancreatic tissue was followed by PanNET in one patient, and in the last patient, the biopsy was positive for PDAC twice. One patient had a biopsy of two separate lesions and was therefore studied twice.

The outcomes of the FNA in individual patients are shown in **Table 6**. In 24 patients, FNA diagnosis was a PanNET; one patient had two FNAs of a different PanNET. In 9 patients, this was subsequently confirmed after operative resection, and 15 patients had imaging follow-up confirming the presence of a PanNET. Six patients had either a normal tissue, a cyst, or an unrepresentative FNA; all were considered to have a PanNET at follow-up with the FNA most likely being a sampling error—two underwent resection and four had imaging follow-up. Of the four patients with PDAC according to the FNA, two patients finally had a PDAC and two a PanNET. In one patient, the FNA and the following surgical resection specimen showed a PDAC. In one patient, two biopsies of the same lesion were positive for PDAC, which was the cause of death shortly after. In one patient with FNA positive for PDAC, the tumor in the operative resection specimen appeared to be a PanNET. In one patient, the FNA was initially positive for PDAC; revision of the specimen during follow-up changed the conclusion into a PanNET. The median age of the three patients with a final diagnosis of a PDAC was 68.8 years (range, 61.9–75.5).

**Table 6:** FNA results in individual patients.

Pt no.	PanNET no.	CT/MRI before FNA	Size PanNET^|^ (mm)	Year FNA	Time 1st positive imaging until FNA (years)	FNA result	Time FNA until resection (years)	Surgery outcome	Follow-up
1	1	Positive	50	2001	0	PanNET	0.5	PanNET WHO G2	
2	2	Positive	10.5	2004	2.3	PanNET			PanNET
	3	Positive	5	2004	2.3	PanNET			PanNET
3	4	Positive	23	2009	8.3	PanNET			PanNET
		Positive	20	2013	13	PanNET			
4	5	Positive	24	2009	1.5	PanNET	0.3	PanNET WHO G1	
5	6	Positive	21	2012	7.8	PanNET			PanNET
6	7	Positive	40	1995	0	Normal pancreas			PanNET
7	8	Positive	8	2010	3.5	PanNET			PanNET
8	9	Positive	13	2009	4	PanNET			PanNET
9	10	Positive	22	2007	0.3	Normal pancreas			PanNET
		Positive	22	2008	0.8	PanNET			
10	11	Positive	13	2007	0.3	PanNET			PanNET
11	12	Negative	13.7	2009	NA^*^	PanNET			PanNET
12	13	Positive	13	2005	0.3	PanNET	0	PanNET WHO G1	
13	14	Positive	15	2016	17	PanNET			PanNET
14	15	Positive	160	2007	0	PanNET	0.5	PanNET WHO G1	
15	16	Positive	54	2000	1	PDAC			PDAC
		Positive	54	2001	1.3	PDAC			
16	17	Positive	60	1994	4.5	PDAC			PanNET
17	18	Positive	60	2000	0	PanNET			PanNET
18	19	Positive	14	2009	0	Normal pancreas/not representative			PanNET
19	20	Negative	15.1	2009	NA^*^	PanNET			PanNET
20	21	Positive	10	2010	0.3	PanNET	4.5	PanNET WHO G1	
21	22	Positive	24.4	2007	0.3	Cyst	0.5	PanNET WHO G1	
22	23	Positive	40	2016	7.5	PDAC	0	PDAC	
23	24	Positive	20	2004	9.8	PanNET			PanNET
24	25	Negative	Unknown	2004	NA^*^	Normal pancreas	8.8	PanNET WHO G1	
25	26	Positive	25	2014	4.5	PanNET	0.9	PanNET WHO G2	
26	27	Positive	Unknown	2013	0.5	Not representative			PanNET
27	28	Positive	10	2011	0	PDAC	0.3	PanNET WHO G1	
28	29	Positive	60	2013	0	PanNET			PanNET
29	30	Positive	21	2017	3.3	PanNET	0.2	PanNET WHO G1	
30	31	Positive	8	2016	0.8	PanNET	0	PanNET WHO G1	
31	32	Positive	7	2016	0.3	PanNET	0	PanNET WHO G1	
32	33	Negative	6	2017	NA^*^	PanNET			PanNET
33	34	Negative	10	2015	NA^*^	Cyst			PanNET

^*^EUS first positive imaging study.

^|^Based on CT, MRI, or EUS.

CT, computed tomography; EUS, endoscopic ultrasonography; FNA, fine needle aspiration; MRI, magnetic resonance imaging; NA, not applicable; PanNET, pancreatic neuroendocrine tumor; PDAC, pancreatic ductal adenocarcinoma.

## Discussion

The present study shows an increase in the use of pancreatic imaging in patients with MEN1 over the past three decades and a shift in imaging modality towards MRI. The diagnostic accuracy of the pancreatic imaging in this population-based cohort of patients with MEN1 was high. The diagnostic accuracy of MRI is excellent and exceeds that of CT, particularly for NETs of the pancreatic head. Routine histopathological confirmation of PanNETs can be considered to have a limited added value over repeat imaging for the diagnosis of MEN1-related PanNETs.

The imaging-based screening program is quintessential to enable timely diagnosis of MEN1-related PanNETs. The present study shows the tremendous cumulative burden of pancreatic imaging in MEN1, as 70–80 scans per 100 patients with MEN1 are annually performed, which does not include imaging of the pituitary gland and lungs. In addition, these observed numbers are an underestimation of all pancreatic imaging, since each modality was only captured once per quarter. The number of imaging studies has substantially increased especially after the first MEN1 clinical practice guidelines in 2001 (3). A substantial shift towards MRI was observed. Over one-third of the index scans was documented as positive in the most recent decade. After the DMSG MEN1 database initiation in 2008, the Dutch collaboration also led to nationwide protocols aiming to standardize clinical practice processes. The high number of scans showing a PanNET could be attributed to the increased diagnostic accuracy of new scanners, increased experience of endosonographers, the duration of follow-up and patients’ age, and the adjustments of imaging protocols.

It is currently unclear whether histopathological confirmation of PanNET is needed in MEN1 after a radiological diagnosis. This study shows that the overall PPVs of MRI, CT, and EUS combined were 88.9% (76.0–95.6) for the head and 92.0% (85.3–96.0) for the body/tail, indicating that routine histopathology or FNA could potentially add a maximum of 11.1% (4.4–24.0) and 8.0% (4.0–14.7) on top of imaging studies for the head and body/tail, respectively. To be used as an add-on, the diagnostic accuracy must improve the existing diagnostic pathway (20). Of the 10 biopsies that were not considered as a PanNET by FNA, eight were considered as PanNET after surgical resection or follow-up. Two of the four patients with PDAC in the FNA were incorrectly considered as PDAC. These numbers indicate that routine FNA confirmation is not necessary to accurately diagnose a pancreatic lesion in MEN1 as a PanNET. However, it was unknown in how many patients did FNA change clinical management. Considering the high prior probability for PanNETs in MEN1 as compared with sporadic PanNET, a positive FNA will not add much information to the diagnostic process, whereas a negative FNA is most often due to sampling error. For newly diagnosed lesions, biopsies could be considered selectively based on age and atypical radiological characteristics to identify the rare cases of PDAC. PanNETs generally present as homogeneous and well-circumscribed solid or cystic hypervascular tumors. In contrast, CT features of PDAC include a hypo- or iso-attenuating mass during the pancreatic phase and secondary signs such as ductal dilatation, a cutoff of the main pancreatic duct, distal pancreatic atrophy, bile duct dilatation or irregular pancreatic contours, and possible vascular encasement (21, 22). On MRI, PDAC appears hypo- to isointense on post-contrast T1-weighted MRI, and the same secondary signs apply (21). MRI features for PanNETs include hyperintense and hypervascular tumors on T2-weighted images and contrast-enhanced T1 images. MRI is superior in assessing the morphology of the pancreatic duct and possible cystic components of the pancreatic lesion. In this study, three patients, all of whom were older than 60 years, were considered to have a PDAC. PDAC in MEN1 has been rarely reported in the literature, including one case report (23), a prevalence and cause of death in 0%–0.3% in multicenter (6, 24) and single center (25–27) studies, and 0% in a literature review (25) including 1,613 patients.

The sensitivity in previous studies ranged from 54% to 81% for CT and 74% to 88% for MRI (8). Within our cohort with index imaging between 2010 and 2017, the sensitivity of the pancreatic body/tail was on the high end of the percentages reported in the literature. These higher percentages are likely a consequence of including imaging studies from recent years compared to other studies. However, for the pancreatic head, sensitivity of especially CT was lower. In the present study, index scans were analyzed, whereas in studies included in the systematic review, not necessarily index scans were used or surgical cohorts were analyzed, which could influence the sensitivity (8). In addition, the outcomes of our cohort indicate that the diagnostic accuracy of MRI is high, irrespective the location of the lesion.

The choice for an imaging modality is not solely guided by the diagnostic accuracy to detect PanNETs but also based on its ability to detect lymph node and liver metastasis, the precision of tumor size estimation, invasiveness, local availability, side effects, and costs. EUS lacks the ability to detect distant metastases. Somatostatin receptor imaging and CT are associated with ionizing radiation. The exact role of PET/CT in the setting of MEN1 is still unknown; however, somatostatin receptor or glucagon-like peptide-1 receptor positron emission tomography (PET)/CT should not be regarded as first-line screening modality but could be subsequently used to detect metastases or to localize insulinomas (8, 28). In this respect, a systematic review concluded that PET/CT could be subsequently added for non-functioning PanNETs larger than 10 mm to detect metastases (8). The added value of somatostatin receptor PET/CT was recently shown, however, in screened patients without a previous PanNET; PET/CT was positive in 90.9% of patients, and MRI was positive in 92.3% (29). In this study, more metastases were detected on PET/CT, and therefore, PET/CT could be additionally used for (preoperative) staging (29). MRI can accurately estimate tumor size, the main prognostic factor (24, 30, 31). Although no preference is reported within the guidelines, this study shows that clinical decision making tends to prefer MRI in the Netherlands. Considering the diagnostic accuracy, patterns in daily practice, and the consequences of undesirable radiation exposure in a hereditary disease with necessary lifelong radiological follow-up, MRI should be considered the preferred non-invasive imaging modality.

In this paper, we focus on the diagnostic value of FNA; however, it is important to consider that FNA could potentially contribute to risk stratification and thereby to personalized follow-up and treatment. This is currently not part of standard clinical practice in MEN1. There is a need for novel non-invasive markers for risk stratification such as liquid biopsies or imaging-based risk stratification (32).

The major strength of our study is the population-based cohort comprising patients from eight academic institutions taking care of more than 90% off all Dutch MEN1 patients, which subsequently reduces case-mix issues and provides an accurate disease prevalence. Previous studies mainly were single-center studies, with selected patient cohorts (e.g., surgically treated patients), and non-invasive imaging modalities were only compared in <10 patients (8). Appropriate methods to deal with differential verification such as an alternative reference standard with stratified analyses were performed (15). By adapting an alternative reference standard, the study population included all potential patients with a PanNET and not specifically those who underwent operative resection, leading to a representative cohort. This allowed representative contingency tables and calculation of all corresponding diagnostic accuracy measures, whereas former studies mainly reported sensitivity. In addition, this study was the first to compare MRI and CT within a recent cohort. Subgroup analyses according to time periods were performed to account for the increased diagnostic accuracy over time. The main study limitations are adherent to the database design, including its retrospective nature, including only one tumor from the pancreatic head and body/tail, the data collection by multiple investigators, and the data collection from daily practice including different scanners and different scan protocols. Nevertheless, a stringent protocol was adhered to in order to realize uniform data collection, and the number of data collectors was minimized and low, taking the number of imaging studies into account (13). Only a prospective comparative study including MRI, CT, and EUS plus FNA in all patients and standardized and blinded reading and reporting of imaging reports would overcome some of these limitations. In this respect, The European Neuroendocrine Tumor Society has only published protocols for standardized reporting of imaging for PanNETs to improve uniformity and potentially reduce underreporting (33). Nevertheless, the present analysis, including multiple readers from each participating hospital, without a centralized review by a dedicated MEN1/NET team, reflects current daily clinical practice. In addition, at the time of reading imaging studies, radiologists, gastroenterologists, and pathologists were not aware that their contributions were used for scientific research, making a Hawthorne effect unlikely. Furthermore, all participating centers were academic hospitals. Since the imaging studies were captured from clinical practice, the time between the index test and reference standard could vary and was not adjusted for in the analysis. The number of FNAs was relatively low; nevertheless, the number of patients undergoing surgical resection was substantial, and therefore, histopathology was obtained in more than 25% of the cohort. The impact of EUS-guided FNA on clinical decision making in individual patients is unknown due to the retrospective nature of the database. For radiological follow-up as reference standard, no adjustment was done for which scan was performed first or whether follow-up was performed by CT or MRI. Nevertheless, we suggest that the decision for the choice of the first scan was influenced by the treating physician and hospital and not necessarily by other factors affecting the chance of having a PanNET. In addition, a cutoff of 5 mm within 3 years was applied to consider which increased the number of false negative studies.

In conclusion, the use of pancreatic imaging studies (CT, MRI, and EUS) in MEN1 has substantially increased over the past decades, and a shift towards MRI was observed. The overall diagnostic accuracy of imaging for PanNET in MEN1 is high, and the incidence for PDAC was nil in patients younger than 60, so routine pancreatic biopsies will likely add little additional information for the differential diagnosis of pancreatic cancer. The potential use of FNA in prognostication and treatment planning is beyond the scope of this work. Diagnostic accuracy measures of MRI are excellent. Considering the disadvantages of CT and the high diagnostic accuracy of MRI and its predominant use, MRI should be advocated as the preferred (non-invasive) modality for the detection of MEN1-related PanNETs.

## Author's note

The abstract has been presented at the 2022 European Congress of Endocrinology (ECE) in Milan and has received a Young Investigators Award.

## Data availability statement

The datasets presented in this article are not readily available because of the sensitive nature of the data collected for this study, the authors cannot make the data publicly available. Requests to access the datasets should be directed to Carolina R.C. Pieterman, c.r.c.pieterman@umcutrecht.nl.

## Ethics statement

This study was reviewed and approved by METC UMC Utrecht. Written informed consent to participate in this study was provided by the participants or their legal guardian as appropriate.

## Author contributions

D-JB: study design, acquisition of data, statistical analysis and interpretation of data, drafting, and final approval of the manuscript. CP: study design, design of data collection protocol, acquisition of data, critical revision, and final approval of the manuscript. FW: study design, critical revision, and final approval of the manuscript. AV: study design, critical revision, and final approval of the manuscript. WH: study design, critical revision, and final approval of the manuscript. OD: study design, critical revision, and final approval of the manuscript. WZ: study design, critical revision, and final approval of the manuscript. MD: study design, critical revision, and final approval of the manuscript. PB: study design, critical revision, and final approval of the manuscript. BH: study design, critical revision and final approval of the manuscript. IB: study design, critical revision, and final approval of the manuscript. MV: study design, interpretation of data, critical revision and final approval of the manuscript, and study supervision. GV: study design, interpretation of data, critical revision and final approval of the manuscript, and study supervision. All authors contributed to the article and approved the submitted version.

## Conflict of interest

The authors declare that the research was conducted in the absence of any commercial or financial relationships that could be construed as a potential conflict of interest.

## Publisher’s note

All claims expressed in this article are solely those of the authors and do not necessarily represent those of their affiliated organizations, or those of the publisher, the editors and the reviewers. Any product that may be evaluated in this article, or claim that may be made by its manufacturer, is not guaranteed or endorsed by the publisher.
