# Supplementary material for: Diagnosing pancreatic neuroendocrine tumors in patients with multiple endocrine neoplasia type 1 in daily practice
Source: Front Endocrinol (Lausanne). 2022 Oct 7;13:926491. doi: 10.3389/fendo.2022.926491 (PMC9585192; doi:10.3389/fendo.2022.926491)
Supplement: Supplementary file 1 [file DataSheet_1.docx]

Supplementary Table 1. Characteristics of imaging studies.

| **Supplementary Table 1. Characteristics of imaging studies.** | | | | |
| --- | --- | --- | --- | --- |
|  | **All imaging** | **MRI** | **CT** | **EUS** |
| Total no of scans (%) | 3477 | 1818 (52.3%) | 1291 (37.1%) | 368 (10.6%) |
| No of scans per patient, median [IQR, range] | 7 [3-11, 0-32] | 4 [2-7, 0-19] | 2 [0-4, 0-29] | 0 [0-1, 0-14] |
| PanNET, no (%) | 1845 (53.1%) | 959 (52.8%) | 560 (43.4%) | 326 (88.6%) |
| PanNET head, no (%) | 932 (26.8%) | 452 (48.5%) | 251 (19.4%) | 229 (62.2%) |
| PanNET body/tail, no (%) | 1471 (42.3%) | 773 (42.5%) | 408 (31.6%) | 290 (78.8%) |
| Total scans performed, no (%)  1990-1999  2000-2009  2010-2017 | 255 (7.3%)  1237 (35.6%)  1985 (57.1%) | 98 (38.4%)  502 (40.6%)  1218 (61.4%) | 153 (60.0%)  605 (48.9%)  533 (26.9%) | 4 (1.6%)  130 (10.5%)  234 (11.8%) |
| Index scans positive (%)  1990-1999  2000-2009  2010-2017 | 28/71 (39.4%)  62/206 (30.1%)  44/119 (37.0%) | 12/31 (38.7%)  17/86 (19.8%)  28/83 (33.7%) | 16/40 (40.0%)  44/119 (37.0%)  16/36 (44.4%) | -  1/1 (100%)  - |
| All scans performed per patient were included.  Abbreviations: *CT* computed tomography, *EUS* endoscopic ultrasound, *IQR* interquartile range, *MRI* magnetic resonance imaging, *PanNET* pancreatic neuroendocrine tumor | | | | |

Supplementary Table 2. Percentage positive index scans stratified by age category.

| **Supplementary Table 2. Percentage positive index scans per study period stratified by age category.** | | | | | | | | |
| --- | --- | --- | --- | --- | --- | --- | --- | --- |
|  |  | **Age category (years)** | | | | | | |
|  | **<20**  **(n = 61)** | **20 – 29**  **(n = 69)** | **30 – 39**  **(n = 75)** | **40 – 49**  **(n = 81)** | **50 – 59**  **(n = 59)** | **60 – 69**  **(n = 44)** | **>70**  **(n = 7)** | **All**  **(n = 396)** |
| **1990 – 1999** | 25.0%  (1/4) | 23.1%  (3/13) | 38.9%  (7/18) | 50.0%  (9/18) | 55.6%  (5/9) | 25.0%  (2/8) | 100%  (1/1) | 39.4%  (28/71) |
| **2000 – 2009** | 29.6%  (8/27) | 17.5%  (7/40) | 34.1%  (15/44) | 32.6%  (15/46) | 36.4%  (8/22) | 33.3%  (8/24) | 33.3%  (1/3) | 30.1%  (62/206) |
| **2010 – 2017** | 23.3%  (7/30) | 43.8%  (7/16) | 30.8%  (4/13) | 35.3%  (6/17) | 35.7%  (10/28) | 66.7%  (8/12) | 66.7%  (2/3) | 37.0%  (44/119) |
| **All** | 26.2%  (16/61) | 24.6%  (17/69) | 34.7%  (26/75) | 37.0%  (30/81) | 39.0%  (23/59) | 40.9%  (18/44) | 57.1%  (4/7) | 33.8%  (134/396) |
| Data denote the percentage of positive scans, number of positive scans and total number of scans.  For each patient their exact age at the time of the imaging study was calculated. Patients were subsequently categorized into age groups at the time of the index scan. | | | | | | | | |

Supplementary Table 3. Contingency tables stratified by reference standard.

| **Supplementary Table 3. Contingency tables stratified by reference standard.** | | | | | | | |
| --- | --- | --- | --- | --- | --- | --- | --- |
| **Reference standard = pathology** | | | | **Reference standard = imaging follow-up** | | | |
|  | **PanNET (no, %)** | **No PanNET (no, %)** |  |  | **PanNET (no, %)** | **No PanNET (no, %)** |  |
| **Pancreatic head** | | | Total | **Pancreatic head** | | | Total |
| Index PanNET | 14 (70) | 0 (0) | 14 (70.0) | Index PanNET | 26 (59.1) | 5 (1.6) | 31 (8.7) |
| Index no PanNET | 6 (30) | 0 (0) | 6 (30.0) | Index no PanNET | 18 (40.9) | 309 (98.4) | 327 (91.6) |
| Total | 20 (100) | 0 (0) | 20 (100) | Total | 44 (12.3) | 313 (87.7) | 357 (100) |
| **Pancreatic body/tail** | | | Total | **Pancreatic body/tail** | | | Total |
| Index PanNET | 56 (80.0) | 0 (0) | 56 (80.0) | Index PanNET | 47 (65.3) | 9 (3.8) | 56 (18.2) |
| Index no PanNET | 14 (20.0) | 0 (0) | 14 (20.0) | Index no PanNET | 25 (34.7) | 226 (96.2) | 251 (81.8) |
| Total | 70 (100) | 0 (0) | 70 (100) | Total | 72 (23.5) | 235 (76.5) | 307 (100) |
| In the 2x2 tables percentages are column percentages. The row “total” has row percentages, the column “total” has column percentages.  Abbreviations: *PanNET* pancreatic neuroendocrine tumor | | | | | | | |

Supplementary Table 4. Diagnostic accuracy measures stratified by reference standard.

| **Supplementary Table 4. Diagnostic accuracy measures according to reference standard.** | | | | | | |
| --- | --- | --- | --- | --- | --- | --- |
|  | **Sensitivity** | **Specificity** | **PPV** | **NPV** | **Positive LR** | **Negative LR** |
| **Pancreatic head** | | | | | | |
| **Pathology** | 70.0 (47.9 – 85.7) | NA | 100 (74.9 – 100) | 0 (0.0 – 44.3) | NA | NA |
| **Imaging** | 59.1 (44.4 – 72.3) | 98.4 (96.2 – 99.4) | 83.4 (66.9 – 93.4) | 94.5 (91.4 – 96.5) | 37.1 (15.0 – 91.6) | 0.42 (0.29 – 0.59) |
| **Pancreatic body/tail** | | | | | | |
| **Pathology** | 80.0 (69.1 – 87.8) | NA | 100 (92.3 – 100) | 0 (0.0 – 25.1) | NA | NA |
| **Imaging** | 65.3 (53.7 – 75.3) | 96.2 (92.8 – 98.1) | 83.9 (72.0 – 91.5) | 90.0 (85.7 – 93.2) | 17.0 (8.8 – 33.1) | 0.36 (0.26 – 0.50) |
| 95% CIs are given in parentheses.  Abbreviations: *LR* likelihood ratio, *NPV* negative predictive value, *PPV* positive predictive value | | | | | | |
